# Three-Arm Registry-Based Comparison of Trans-Inguinal-Pre-Peritoneal, Laparoscopic, and Lichtenstein Techniques for Scrotal Hernia Repair

**DOI:** 10.3389/jaws.2025.13993

**Published:** 2025-07-29

**Authors:** J. F. Gillion, M. Soler, A. Mettoudi, A. Lamblin, A. C. Couchard, O. Oberlin, J. P. Cossa, N. Maillot, F. Jurczak, The Club-Hernie Members

**Affiliations:** ^1^ Department of General Surgery, Ramsay Sante Hôpital Privé d’Antony, Antony France; ^2^ Department of General Surgery, Polyclinique Saint Jean, Cagnes-sur-Mer, France; ^3^ Department of General Surgery, Centre Hospitalo-Universitaire de Nice, Nice, France; ^4^ Department of General Surgery, Hôpital Privé La Louvière, Lille, France; ^5^ Department of General Surgery, Clinique Turin, Paris, France; ^6^ Department of General Surgery, CMC Bizet, Paris, France; ^7^ Department of General Surgery, Clinique du Parc, Cholet, France; ^8^ Department of General Surgery, Clinique Mutualiste, Saint Nazaire, France

**Keywords:** groin hernia repair, minimal invasive open preperitoneal repair, scrotal hernia, registry-based comparative study, transinguinal preperitoneal technique

## Abstract

**Background:**

Studies on minimal invasive open preperitoneal techniques performed in scrotal hernia repair are very scarce.

**Methods:**

We conducted a comparative study based on the prospectively collected data of the “Club-Hernie.” A scrotal hernia was defined as an inguinal hernia which has descended into and causes any distortion of the scrotum. Giant inguinal hernias were not included.

**Results:**

A total of 3,043 scrotal hernias repairs, performed from 01/09/2011 to 30/04/2023, met the inclusion criteria. The late results of 395 Trans-Inguinal-Pre-Peritoneal (TIPP/MOPP), compared with those of 1038 Lichtenstein and those of 1610 laparoscopic (TEP/TAPP) repairs were globally similar. At a median follow-up of 2 years, no significant difference was found between the three groups regarding the rate of identified recurrences (0.6% vs. 0.6% vs. 0.7%; p=0.9191; p=0.7435) and the prevalence of severe CPIP (0.6% vs. 0.4% vs. 0.7%; p=0.6772; p=0.7300, respectively for TIPP, Lichtenstein and TEP/TAPP). Each technique, though, showed some benefits and drawbacks. Laparoscopic repairs, used in this series in less complex patients (lower number of ASA 3-4 patients and/or patients on anticoagulants) and hernias (lower rates of L3/M3 defects), provided a better nerve preservation (nerve resection /= III) postoperative complications and a high rate of day surgery (69.9%). The hernia sac was completely resected in 64% of cases without injury of the spermatic cord nor need for a unilateral orchidectomy. Probably due to preoperative tailoring, the Lichtenstein group significantly collected many of the most complex patients (ASA3-4: 31.8%; anticoagulant therapy: 23.4%) and the most symptomatic hernias (severe preoperative pain: 17.5%). Lichtenstein was not only a default technique but also a fallback procedure: Fifteen (40.5%) of the 37 conversions occurring in laparoscopic or TIPP techniques ended up in a Lichtenstein technique.

**Conclusion:**

This study shows that TIPP is feasible, safe and effective in scrotal hernias, providing results close to those of laparoscopic techniques. Thus, TIPP appears as a valid alternative when the aim is to elect both a preperitoneal repair and a minimal invasive open route. Having the choice of effective techniques may help in tailoring the treatment of these so particular types of groin hernias.

## Introduction

Groin hernias represent a significant global health burden, with over 20 million hernia repairs performed annually worldwide. According to a recent systematic review and management guidelines for scrotal inguinal hernias recently published by Tran et al. [1], scrotal hernias account for approximately 6% of all hernia repairs in high-income countries, with this figure potentially rising to 67% [2] in low-income countries. Irrespective of available access to surgical intervention, scrotal hernias present significant challenges even to experienced surgeons because they are associated with higher morbidity and mortality rates compared to non-complex groin hernia repairs [3]. Instead of the classic definition of a scrotal hernia: “Inguinal hernia which has descended into and causes any distortion of the scrotum” the guidelines working group [1] suggests using a more precise definition: S1 (upper third of the thigh), S2 (middle third of the thigh) and S3 (lower third of the thigh/patella). S(IR) is used to denote an irreducible scrotal hernia.

These guidelines discuss various types of repairs, but open-preperitoneal techniques are not included because of the lack of published studies on using these methods for scrotal hernia repairs.

Therefore, we conducted two successive studies, based on the data of the Club-Hernie Registry [4]:

The initial study, which was monocentric, demonstrated that minimally invasive open pre-peritoneal techniques could achieve similar outcomes in both scrotal and non-scrotal hernia repairs [5]. This led to the present multicentric study with the aim of assessing the feasibility, safety, and effectiveness of minimally invasive open preperitoneal techniques for scrotal hernias compared with the Lichtenstein and laparoscopic techniques.

## Materials and Methods

This retrospective cohort study was conducted in accordance with the STROBE [6] statements and the recommendations of the European Registry of Abdominal Wall Hernias working group [7].

### Study Design

We performed a comparative study using registry data that was prospectively collected in the “Club Hernie” database. Out of all the consecutive inguinal hernia repairs recorded from 01 September 2011 to 30 April 2023, we identified scrotal hernia repairs as defined below.

The exclusion criteria were as follows: patients under 18 years old, patients incorrectly registered as female patients, recurrent hernias, emergency surgery, repairs performed after 30 April 2023, and missing day 30 postoperative outcomes.

Five techniques were included in the study: TIPP (Trans-Inguinal-Pre-Peritoneal), MOPP (Minimal Open Pre-Peritoneal), TEP (Totally Pre-Peritoneal), TAPP (Trans-Abdominal-Pre-Peritoneal) and Lichtenstein. These were clustered in three study groups: TIPP group (TIPP/MOPP), the laparoscopic group (TEP/TAPP) and the Lichtenstein group. These were compared head-to-head. The other techniques were not studied.

### Studied Surgical Techniques

The Trans-Inguinal-Pre-Peritoneal (TIPP) repair technique has already been widely described in the literature [8–10]. In brief, it is a minimally invasive preperitoneal open technique: after minimal inguinal dissection, preservation of the inguinal nerves, and possible resection of the hernia sac, the preperitoneal space is entered, and a flat mesh is inserted through the deep inguinal ring, in between the peritoneum and the abdominal wall, thus totally pre-peritoneally and widely covering the myo-pectineal area.

The MOPP technique [11], is a variant of the TIPP technique, inspired by the Ugahary technique [12], which uses specific long blade dissectors and retractors to dissect and deploy the mesh through the deep inguinal ring. TIPP and MOPP were grouped together for the analysis.

TAPP, TEP and Lichtenstein techniques are standard techniques, which are well known worldwide and do not require any additional description. TEP and TAPP were grouped together for the analysis.

### Club Hernie Registry

The registry is compliant with the European General Data Protection Regulation (GDPR) [13]. The registry-based design of the study guarantees that all data are de-identified and collected with a patient “non-opposition” agreement. It is also compliant with the national ethical standards of the French “Commission Nationale de l’Informatique et des Libertés” (CNIL) (registration number: 1993959v0).

### Follow-Up, PROM Assessment and Late Complication Identification

Each Club Hernie member registered themselves with the pre-, intra-, and day 30 postoperative outcomes of their patients in the online database. Data entry was finalised during the first month (M1) routine clinical visit by the operating surgeon. An optional third-month visit (M3) was scheduled only if any issues were identified at M1. Subsequently, an independent clinical research assistant (CRA) managed the 1-, 2, and 2–5-year follow-up of the patients. This involved using a standardised phone Patient Reported Outcome Measure (PROM) questionnaire, which has been used in our clinical studies since 1999 [14]. During these follow-up interviews, patients were systematically asked about rehospitalisation (either at the same hospital or at a different hospital), reoperation and the causes thereof, confirmed hernia recurrence (whether through reoperation, report of ultrasound or CT scan, and/or surgeon visit), suspected recurrences (identified via the PINQ-Phone questionnaire [15, 16], localised bulging and/or local pain), late abscesses, chronic mesh fistula, mesh removal, and other late complications such as bowel obstruction.

Following five failed attempts to contact the patient on different occasions, they were deemed as lost to follow-up. If there was any deviation from the expected recovery process, scheduling an appointment at the surgeon’s office was highly advised. Their entries were, thus, recorded in tabs dedicated to the surgeon. A few surgeons recommended periodic clinical visits during the follow-up period.

### Definitions of the Studied Variables

The following hernias were characterised preoperatively: i. Femoral hernia; ii. Inguinal hernia (limited to the groin area); iii. “Inguinofunicular” hernia (descending along the spermatic cord but not extending to the scrotum); iv. Scrotal hernia (inguinal hernia that had descended into the scrotum and caused any type of distortion of the scrotum and intra-operatively L0 to L3, M0 to M3, F0 to F3 according to the European Hernia Society groin hernia classification). On day 30, postoperative complications were clustered as follows [17]: i. General complications; ii. Surgical site occurrences (SSOs) including superficial or periprosthetic SSI (Surgical Site Infection) and superficial or periprosthetic SSO non-SSI (seromas); and iii. Organ space (surgical) complications. In cases of concurrent complications, the Clavien-Dindo [18] grading system was applied to the worst complication.

Chronic postoperative inguinal pain (CPIP), defined as pain lasting more than 3 months, was evaluated during follow-up using a 4-scale (no pain, mild pain, moderate pain, or severe pain) VRS (Verbal Rating Scale) and compared with the preoperative status collected at baseline.

Hernia recurrences were categorised as reoperated recurrences and recurrences not reoperated on but confirmed by CT scan, ultrasound, or surgical clinical visit.

### Outcomes of Interest

The feasibility of TIPP was evaluated by the rate of conversion of TIPP into another technique. Safety was based on early and late complications, and effectiveness was assessed by comparing the recurrence rate with that of other studied techniques.

### Descriptive Statistics

Discrete variables were presented as absolute numbers with percentages. Continuous variables were displayed as the median and interquartile range (IQR). Discrete variables were compared using the Chi-square or Fisher’s exact test, and continuous variables were examined using Student’s T test. A p value <0.05 was considered statistically significant. Statistical tests were carried out using the Sorbonne University tool^1^.

## Results

**FIGURE 1:**
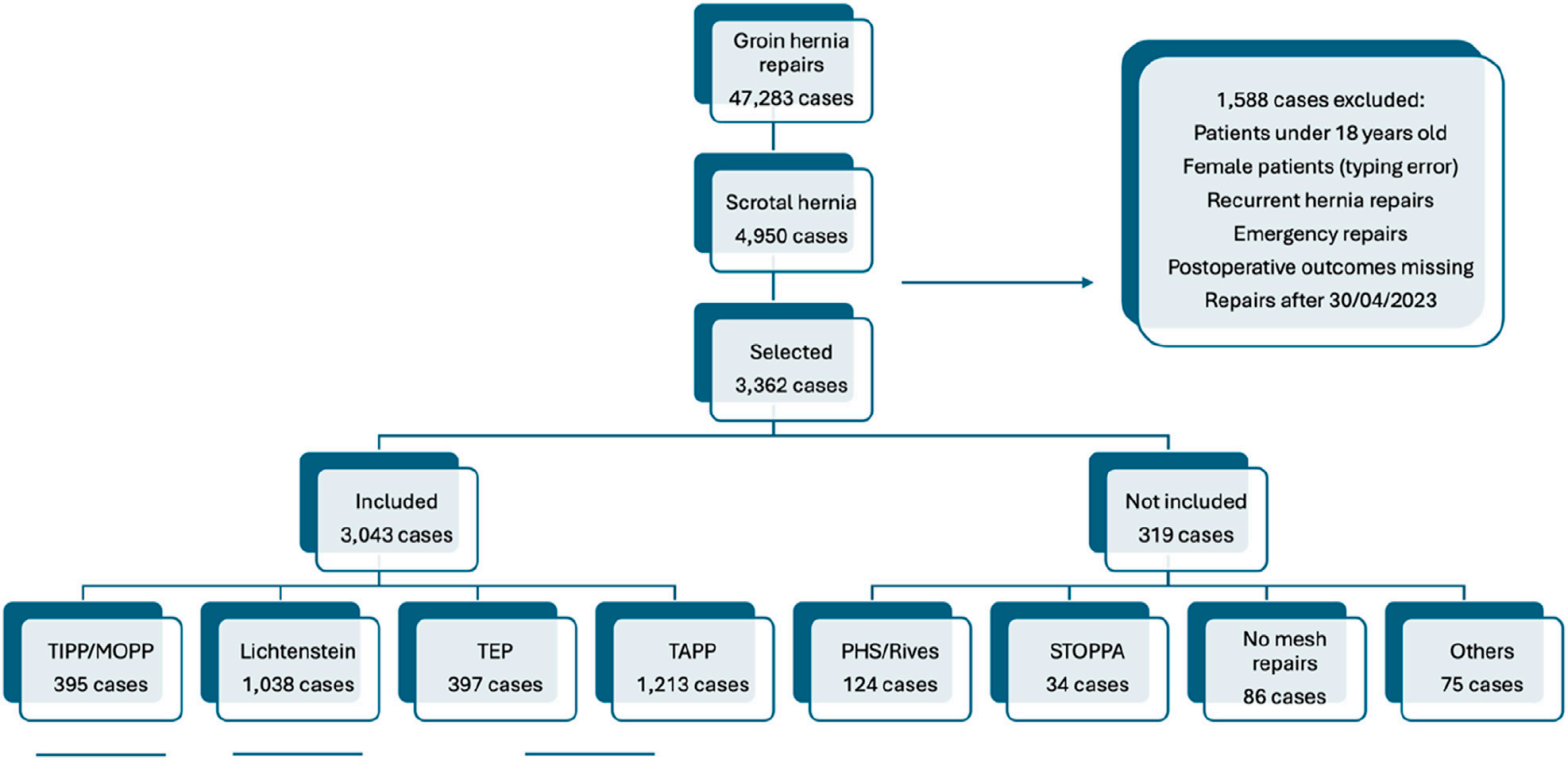
Prisma flowchart. TIPP: Trans-inguinal pre-peritoneal; MOPP: minimal invasive open pre-peritoneal; Lichtenstein: Lichtenstein technique; TEP: totally extraperitoneal; TAPP: trans-abdominal pre-peritoneal.

From 01 September 2011 to 30 April 2023, a total of 47,283 groin hernia repairs were prospectively registered in the Club Hernie database. Of these, 4,950 were scrotal hernias, of which 3,362 (7%) met the inclusion criteria (L 1). The present comparative study focused on four main techniques (3,043 repairs), which were clustered into three groups: TIPP, Lichtenstein, and laparoscopic techniques (TEP and TAPP). The remaining 319 repairs, less frequently performed, were considered only for an overview of the general distribution of cases (Figure 1; Table 1). No TREPP (Trans-Rectus-Pre-Peritoneal) repairs were registered in our database and the rare Ugahary procedures were categorised as “others.”

**TABLE 1:** Distribution of hernia repairs in scrotal vs. all groin hernia cohorts.

Surgical technique	Scrotal hernias (N = 3,362)	All groin hernias (N = 47,283)	P Value
TIPP/MOPP	395 (11.8)	5,617 (11.9)	0.9
TEP	397 (11.8)	11,697 (24.7)	0.0001
TAPP	1,213 (36.1)	14,760 (31.2)	0.0001
Lichtenstein	1,038 (30.9)	10,085 (21.3)	0.0001
PHS/RIVES	124 (3.7)	984 (2)	0.05
Stoppa	34 (1)	161 (0.3)	0.001
Suture repairs	86 (2.6)	1,067 (2.2)	0.3
Other	75 (2.2)	2,912 (6.2)	0.0001

Values shown are n (%).

TIPP: trans-inguinal pre-peritoneal; MOPP: minimally invasive open pre-peritoneal; Lichtenstein: Lichtenstein technique; TEP: totally extra peritoneal; TAPP: trans-abdominal pre-peritoneal.

The relative rates of using Lichtenstein and TAPP were higher for scrotal hernias than for all groin hernia repairs, while the relative rate was lower for TEP, and the relative rate of TIPP was the same for scrotal hernias as for all groin hernia repairs (Table 1).

The conversion rate for the three techniques was low, given the scrotal nature of the treated hernias. TEP was the technique with the highest conversion rate (Table 2), with 19 out of 416 cases (4.6%) being converted, to TAPP (10 cases), Lichtenstein (7 cases), TIPP (1 case) and other (1 case). The conversion rate for TAPP was 1.2%. Overall, the global conversion rate for all laparoscopic repairs (TEP/TAPP combined) was 2.1% compared to 0.8% for TIPP. Eighteen out of 1,041 (1.7%) Lichtenstein techniques were converted to suture repairs (17 cases) or the Rives-Stoppa technique via a midline laparotomy (1 case). The differences in conversion rates were not statistically significant, except for TEP versus TIPP (4.6% vs. 0.8%; p = 0.0008).

**TABLE 2:** Conversion rates among the different types of scrotal hernia repair.

Technique	Intent-to-treat (N*)	Conversion			As treated (N)
		Into	N	Rate (%)	
TEP	416	TAPP	10		
		Lichtenstein	7		
		TIPP	1		
		Other mesh repair	1		
		Total	19	4.6	397
TAPP	1,218	Lichtenstein	5		
		Other mesh repair	7		
		Suture repair	3		
		Total	15	1.2	1,213
TIPP	397	Lichtenstein	3	0.8	395
Lichtenstein	1,041	Rives-Stoppa	1		
		Suture repair	17		
		Total	18	1.7	1,038
Total					3,043

TIPP: trans-inguinal pre-peritoneal; Lichtenstein: Lichtenstein technique; TEP: totally extra peritoneal; TAPP: trans-abdominal pre-peritoneal.

N^*^ as an ‘Intent to treat’ basis = N ‘As treated’ + N converted–N, coming from a converted other technique.

For example,: TAPP ‘intent to treat’ = 1,213 + 15–10.

Conversion rate = N conversions/N ‘Intent to treat’.

The median age, the median BMI, the rate of diabetes and the rate of active smokers were similar across the three studied groups (Table 3).

**TABLE 3:** Characteristics of the study population.

Characteristic	TIPP (N = 395)	Lichtenstein (N = 1,038)	P value	TIPP (N = 395)	TEP/TAPP (N = 1,610)	P value
Age (years)	75 (63–83)	75 (64–84)	0.6	75 (63–83)	71 (59–79)	0.6
BMI (kg/m^2^)	25 (23–27)	25 (23–28)	1	25 (23–27)	25 (23–28)	1
ASA 1–2	314 (82.4)	702 (68.2)	<0.0001	314 (82.4)	1,347 (83.9)	0.5
ASA 3–4	67 (17.6)	328 (31.8)		67 (17.6)	258 (16.1)	
ASA missing	14	8		14	5	
Personal history of hernia(s)	90 (22.8)	212 (20.4)	0.3	90 (22.8)	309 (19.2)	0.12
Diabetes mellitus	21 (5.3)	82 (7.9)	0.1	21 (5.3)	97 (6)	0.7
Anticoagulant-antiplatelet	75 (19)	243 (23.4)	0.07	75 (19)	229 (14.2)	0.02
Active smoker	77 (19.5)	188 (18.1)	0.6	77 (19.5)	286 (17.8)	0.5
Preoperative PROM (VRS)						
Missing data	10	15		10	34	
No symptoms	66 (17.1)	67 (6.6)		66 (17.1)	225 (14.3)	
Painless disturbances	2 (0.5)	6 (0.6)		2 (0.5)	8 (0.5)	
Any pain	317 (82.3)	950 (92.9)	<0.0001	317 (82.3)	1,343 (85.2)	<0.0001
Mild pain (discomfort)	177 (46)	502 (49.1)		177 (44.8)	952 (59.1)	
Moderate pain	90 (23.4)	268 (26.3)		90 (22.8)	305 (18.9)	
Severe pain	50 (12.7)	179 (17.5)	0.0342	50 (12.7)	84 (5.2)	<0.0001
Impact of pain/discomfort on quality of life						
Missing data	0	9		0	4	
No impact on your daily life	108 (34.1)	241 (25.6)		108 (34.1)	352 (26.3)	
Does not force you to interrupt any ongoing activity	138 (43.5)	357 (37.9)	<0.0001	138 (43.5)	730 (54.5)	<0.0001
Forces you to interrupt some ongoing activities	32 (10.1)	260 (27.6)		32 (10.1)	199 (14.9)	
Forces you to give up some activities	39 (12.3)	83 (8.8)		39 (12.3)	58 (4.3)	
Total	317 (100)	950 (100)		317 (100)	1,343 (100)	

Values shown are median (IQR) or n (%). Percentages were calculated based on non-empty values.

TIPP: trans-inguinal pre-peritoneal; Lichtenstein: Lichtenstein technique; TEP: totally extra peritoneal; TAPP: trans-abdominal pre-peritoneal; IQR: interquartile range; BMI: body mass index; PROM: patient-related outcome measures; VRS: verbal rating scale.

Lichtenstein patients had higher ASA scores, more symptomatic hernias and experienced more severe preoperative pain than others. Patients who received Lichtenstein and TIPP repairs were more likely to be on anticoagulant therapy than those operated on laparoscopically. Compared with those in the TIPP group, patients in the laparoscopic group were less often on anticoagulants and had less often experienced preoperative severe pain or preoperative symptoms that forced them to give up some activities.

Only hernias registered as scrotal were included. Their distribution according to the EHS classification is shown in Table 4. Lateral, medial and/or femoral hernias were combined in 10.3%–22.2% of cases. “Pantaloon hernias” (combining a lateral and a medial defect) were more frequent in the Lichtenstein group. Larger defects (L3 or M3) were less frequent in the laparoscopic group than in the TIPP or Lichtenstein groups (70% vs. 91.4% vs. 92.6%; p < 0.0001). Complete resection of the hernia sac was less frequent in the laparoscopic group than in the TIPP or Lichtenstein groups (15.6% vs.64.0% vs. 67.7%; p < 0.0001).

**TABLE 4:** Intraoperative data.

Intraoperative data	TIPP (N = 395)	Lichtenstein (N = 1,038)	P value	TIPP (N = 395)	TEP/TAPP (N = 1,610)	P value
Hernia type						
Lateral	371 (94.4)	919 (88.7)	<0.0001	371 (94.4)	1,408 (87.5)	0.0088
Medial	60 (15.2)	332 (32.0)		60 (15.2)	361 (22.4)	
Lateral + medial	−42 (10.7)	−230 (22.2)		−42 (10.7)	−165 (10.3)	
Femoral ± inguinal	5 (1.3)	15 (1.5)		5 (1.3)	6 (0.4)	
Missing data	1	2		1	0	
Defect size (EHS classification)						
L1 ± medial	11 (2.9)	40 (4.4)		11 (2.9)	43 (3.1)	
L2	40 (10.8)	139 (15.1)		40 (10.8)	476 (33.8)	
L3	320 (86.3)	740 (80.5)	0.0149	320 (86.3)	889 (63.1)	<0.0001
M1 ± lateral	5 (8.3)	39 (11.8)		5 (8.3)	42 (11.6)	
M2	14 (23.3)	71 (21.4)		14 (23.3)	81 (22.4)	
M3	41 (68.3)	221 (66.8)	0.0716	41 (68.3)	238 (66.0)	0.7152
L3 or M3	361 (91.4)	961 (92.6)	0.5665	361 (91.4)	1,127 (70.0)	<0.0001
Hernia sac						
Completely resected	149 (64.0)	528 (67.7)	0.2868	149 (64.0)	110 (15.6)	<0.0001
Incompletely resected	84 (36.0)	252 (32.3)		84 (36.0)	596 (84.4)	
Drains	NS	NS		NS	NS	
Operating surgeons^a^	11	61		11	56	
Anaesthesia						
Spinal alone	19 (4.9)	164 (15.9)	<0.0001	19 (4.9)	3 (0.2)	<0.0001
GA + laryngeal mask	260 (66.3)	413 (39.9)		260 (66.3)	27 (1.7)	
GA + tracheal intubation	109 (27.8)	436 (42.1)		109 (27.8)	1,549 (97)	
Other types	4 (1)	22 (2.1)		4 (1)	17 (1)	
Missing data	3	3		3	14	
Antibiotic prophylaxis						
Yes	120 (93.8)	371 (96.1)	0.26	120 (93.8)	653 (97.2)	0.0193
No	8 (6.2)	15 (3.9)		8 (6.2)	16 (2.8)	
Missing data	267	652		267	938	
Mesh						
Mesh supplier	2	9		2	10	
Mesh references	8	37		8	50	
Mesh fixation^c^						
Sutures	14 (3.5)	613 (59.6)	<0.0001	14 (3.5)	5 (0.34)	<0.0001
Staples	0	144 (14)		0	701 (47)	
Glue	0	45 (4.4)		0	52 (3.5)	
Auto adhesive or self-gripping	0	112 (10.9)		0	30 (2)	
No fixation at all	381 (96.5)	195 (19)		381 (96.5)	959 (64.3)	
Missing data	3	9		3	118	
Nerve resection						
Ilio-hypogastric	7 (1.8)	178 (17.2)	<0.0001	7 (1.8)	0	0.0003
Ilio-inguinal	14 (3.6)	178 (17.2)		14 (3.6)	0	
Genital branch of GF	2 (0.5)	111 (10.8)		2 (0.5)	2 (0.13)	
Femoral branch of GF	0	0		0	2 (0.13)	
Missing data	4	10		4	11	
Intraoperative technical difficulties						
Missing data	4	11		4	14	
None	322 (82.4)	937 (91.2)	<0.0001	322 (82.4)	1,338 (83.8)	0.4789
Any	69 (17.7)	90 (8.8)		69 (17.7)	258 (16.2)	
In creating the workspace	15 (3.8)	58 (5.7)	0.1685	15 (3.8)	55 (3.5)	0.7114
In unrolling the mesh	10 (2.6)	7 (0.7)	0.0037	10 (2.6)	31 (1.9)	0.4456
In closing the peritoneum	0	3 (0.3)	0.2848	0	24 (1.5)	<0.0001
Peritoneal tears	47 (12)	25 (2.4)	<0.0001	47 (12)	168 (10.5)	0.3994
Injury to the epigastric vessels	5 (1.3)	1 (0.1)	0.0022	5 (1.3)	10 (0.63)	0.1827
Intraoperative orchidectomy	0	1 (0.1)		0	0	
Bladder injury (sutured)	2 (0.5)	1 (0.1)		2 (0.5)	3 (0.2)	
Operating time (min)	45 (36–55)	35 (25–43)	<0.0001	45 (36–55)	30 (25–45)	<0.0001

Values shown are median (IQR) or n (%).

TIPP: Trans-inguinal pre-peritoneal; Lichtenstein: Lichtenstein technique; TEP: totally extra peritoneal; TAPP: trans-abdominal pre-peritoneal; IQR: interquartile range; GF: genital-femoral nerve; GA: general anaesthesia; NS: not specified (not registered.

^a^ Among a total of 68 surgeons participating in the present study, many surgeons performed different types of repairs.

^b^ According to the Herniasurge classification [1].

^c^ The fixation means were often combined.

Sixty-eight operating surgeons participated in this multicentre study, with an even distribution across the studied techniques. In line with a registry setting, the choice of the technique was left to the discretion of the surgeon. TIPP procedures were performed by 11 surgeons, Lichtenstein repairs by 61 surgeons and TEP/TAPP procedures by 56 surgeons, making a total of 128 out of the 68 participating surgeons (Table 4). This indicates that surgeons have not always used the same technique for all their patients, but rather tailored the technique according to the characteristics of their patients and the hernia.

General anaesthesia with tracheal intubation was almost always used for laparoscopic repairs, while a lighter form of general anaesthesia with a laryngeal mask was primarily used for open repairs, especially for TIPP. Spinal anaesthesia was rarely used except for Lichtenstein repairs.

Nerve resection occurred rarely in laparoscopic repairs, and even rarer in TIPP than in Lichtenstein repairs.

Mesh fixation was not used in almost all TIPP cases, in two-thirds of laparoscopies and only in one-fifth of Lichtenstein repairs.

Intraoperative technical difficulties occurred less often in the Lichtenstein group. Intraoperative complications such as bladder injury or unilateral orchidectomy were very rare, ranging from 0.1% to 0.5% across all three groups.

Operating time was shorter for laparoscopies than for Lichtenstein and TIPP procedures.

The prevalence of serious (Clavien-Dindo >/= III) postoperative complications at day 30 was low (Table 5) ranging from 0.3% to 1.7%. Postoperative complications mainly consisted of benign surgical site occurrences. The rare but serious organ space complications were mainly observed in laparoscopic approaches. The differences between groups were not statistically significant.

**TABLE 5:** Day 30 postoperative outcomes.

Outcomes	TIPP (N = 395)	Lichtenstein (N = 1,038)	P value	TIPP (N = 395)	TEP/TAPP (N = 1,610)	P value
Postoperative complications						
General complications	5 (1.3)	35 (3.6)	0.0232	5 (1.3)	16 (1.1)	0.6381
Missing data	6	60		6	29	
Surgical site occurrences						
Superficial SSO non-SSI (seroma)	35 (9.0)	85 (8.7)	0.8692	35 (9.0)	101 (6.4)	0.07
Periprosthetic SSO non-SSI	4 (1.0)	8 (0.8)		4 (1.0)	6 (0.4)	
Superficial SSI	1 (0.3)	8 (0.8)		1 (0.3)	1 (0.1)	
Periprosthetic SSI	0 (0.0)	0 (0.0)		0 (0.0)	1^a^ (0.1)	
Missing data	5	60		5	27	
Organ space complications						
Bowel obstruction	0 (0.0)	0 (0.0)		0 (0.0)	3 (0.2)	
Peritonitis	0 (0.0)	0 (0.0)		0 (0.0)	2^b^ (0.1)	
Vx injury revealed postoperatively	0 (0.0)	0 (0.0)		0 (0.0)	2 (0.1)	
Orchitis	1 (0.3)	5 (0.5)		1 (0.3)	2 (0.1)	
Hydroceles	3 (0.8)	4 (0.4)		3 (0.8)	1 (0.06)	
Early (< day 30) recurrence	0 (0.0)	0 (0.0)		0 (0.0)	3 (0.2)	
Missing data	5	61			8	
Reoperation	1 (0.3)	17 (1.7)	0.0355	1 (0.3)	12 (0.8)	0.2967
Missing data	30	70		30	55	
Mesh removal < day 30	0 (0.0)	0 (0.0)		0 (0.0)	1^a^ (0.06)	
Clavien-Dindo classification						
Missing data	1	0		1	21	
Patient without complications	343 (87.0)	895 (86.2)		343 (87.0)	1,451 (91.3)	
Patient with any complication	51 (13.0)	143 (13.8)	0.6811	51 (13.0)	138 (8.7)	0.010
Grade I/II	50 (12.7)	126 (12.1)		50 (12.7)	125 (7.8)	
Grade III	1 (0.3)	15 (1.5)		1 (0.3)	12 (0.7)	
Grade IV	0 (0.0)	2 (0.2)		0 (0.0)	0 (0.0)	
Grade V	0 (0.0)	0 (0.0)		0 (0.0)	1 (0.1)	
Clavien-Dindo >/ = III	1 (0.3)	17 (1.7)	0.1559	1 (0.3)	13 (0.8)	0.2311
Postoperative pain (VAS)						
Day 1: median (IQR); missing	3 (2–5); 65	2 (1–3); 111	<0.0001	3 (2–5); 65	2 (1–4); 505	<0.0001
Day 8: median (IQR); missing	1 (0–2); 75	1 (0–2); 152	0.9028	1 (0–2); 75	0 (0–1); 557	<0.0001
Day 30: median (IQR); missing	0 (0–0); 100	0 (0–0); 140	0.6748	0 (0–0); 100	0 (0–0); 612	0.0506
Hospital length of stay						
Missing data	7	15		7	36	
Outpatients	271 (69.9)	611 (59.7)	<0.0001	271 (69.9)	1,250 (79.4)	<0.0001
Inpatients	117 (30.1)	412 (40.3)		117 (30.1)	324 (20.6)	

Values shown are the median (IQR) or n (%). Percentages (in italics) were calculated based on non-empty values.

TIPP: trans-inguinal pre-peritoneal; Lichtenstein: Lichtenstein technique; TEP: totally extra peritoneal; TAPP: trans-abdominal pre-peritoneal; IQR: interquartile range; SSO: surgical site occurrence; SSI: surgical site infection; VAS: visual analogue scale.

Clavien-Dindo classification [18]: in cases of combined complications, the CDC, grading (per patient) was calculated based on the worst complication.

Day 1: Day after the surgical procedure.

^a^ Mesh infection leading to an early mesh removal.

^b^ Peritonitis (inadvertent enterotomy).

Outpatient cases were significantly more frequent following laparoscopies than following TIPP, and following TIPP than following Lichtenstein repairs. Early postoperative pain was significantly lower in the laparoscopic group. This difference disappeared during the first postoperative month and at day 30 the median VAS was zero in all three groups.

More than 85% of patients were followed up for a median period of 2 years (Table 6). The follow-up was twice as long for TIPP as for the other two groups. The recurrence rate (prevalence of identified recurrences) was the same (approximately 0.6%) in all three groups.

**TABLE 6:** Follow-up and cumulative late complications.

Complications	TIPP (N = 395)	Lichtenstein (N = 1,038)	P value	TIPP (N = 395)	TEP/TAPP (N = 1,610)	P value
Patients lost to follow-up	42 (10.6)	63 (6.0)	0.0031	42 (10.6)	238 (14.8)	0.0330
Median follow-up time (months)	59 (23–61)	25 (1–60)		59 (23–61)	26 (3–60)	
Complications during follow-up						
Late (>M3) scrotal collection	0	0		0	0	
Late orchitis/testicular atrophy	0	2 (0.2)		0	0	
Late mesh infection	2^a^ ^,^ ^b^ (0.6)	0	0.0705^c^	2^a^ ^,^ ^b^ (0.6)	0	0.4178^c^
Identified recurrences						
Reoperated	1	0		1	2	
Not reoperated but confirmed	1	6		1	8	
Total hernia recurrences	2 (0.6)	6 (0.6)	0.9191	2 (0.6)	10 (0.7)	0.7435
Total late complications	5 (1.4)	10 (1.3)	0.5516	5 (1.4)	10 (0.7)	0.2146

Values shown are the median (IQR) or n (%). Percentages were calculated based on non-empty values. Percentages of late complications were calculated from the number of patients followed.

TIPP: trans-inguinal pre-peritoneal; Lichtenstein: Lichtenstein technique; TEP: totally extra peritoneal; TAPP: trans-abdominal pre-peritoneal; IQR: interquartile range; PROM: patient-reported outcome measures.

^a^ Mesh removal, no hernia recurrence during the following 5 years.

^b^ Patient reoperated (mesh removal unknown) then lost to follow-up after FU1.

^c^ Fisher’s exact test.

Two cases of unilateral testicular atrophy were identified in the Lichtenstein group, and two late mesh infections occurred in the TIPP group.

At the first follow-up questionnaire (Table 7) the prevalence of a relevant (moderate or severe) pain (CPIP) was lower in the TIPP group than in the Lichtenstein group (3% vs. 6.6%; p = 0.0001) and lower in the TIPP group than in the TEP/TAPP group (3% vs. 6.1%; p = 0.0141). The prevalence of severe pain was 0.5% or less in all three groups. Postoperative pain or discomfort higher than at baseline was reported by less than 1% of patients.

**TABLE 7:** PROMs at FU1 and FU2.

	TIPP (N = 395)	Lichtenstein (N = 1,038)	P value	TIPP (N = 395)	TEP/TAPP (N = 1,610)	P value
FU1phone questionnaire						
Patients reached	250 (63.3)	469 (45.2)		250 (63.3)	753 (46.8)	
Late pain (VRS): CPIP						
Missing data	11	28		11	30	
Asymptomatic patients	218 (91.2)	372 (84.4)	0.0117	218 (91.2)	629 (87)	0.0817
Painless disturbances	2 (0.8)	7 (1.6)		2 (0.8)	10 (1.4)	
Any pain	19 (8)	62 (14.1)	0.0189	19 (8)	84 (11.6)	0.1118
Mild pain (discomfort)	11 (5)	33 (7.3)		11 (5)	40 (5.5)	
Moderate pain	8 (3)	27 (6.1)	0.0001	8 (3)	41 (5.7)	0.0141
Severe pain	0 (0.0)	2 (0.5)		0 (0.0)	3 (0.4)	
Postop pain/discomfort > Preop	1 (0.5)	3 (0.6)	1	1 (0.5)	6 (0.7)	1
FU2 phone questionnaire						
Patients reached	152 (38.5)	228 (22)		152 (38.5)	417 (25.9)	
Late pain (VRS): CPIP						
Missing data	0	0		0	0	
Asymptomatic patients	133 (87.5)	203 (89.0)	0.7438	133 (87.5)	377 (90.4)	0.3141
Painless disturbances	3 (2.0)	6 (2.6)		3 (2.0)	3 (0.7)	
Any pain	16 (10.5)	19 (8.3)	0.4746	16 (10.5)	37 (8.9)	0.5538
Mild pain (discomfort)	8 (5.3)	9 (3.9)		8 (5.3)	18 (4.3)	
Moderate pain	7 (4.6)	9 (3.9)	0.6772	7 (4.6)	16 (3.8)	0.7300
Severe pain	1 (0.6)	1 (0.4)		1 (0.6)	3 (0.7)	
Postop pain/discomfort > Preop	0	0	1	0	2 (0.5)	1

Values shown are n (%). Percentages were calculated based on non-empty values.

PROM: patient-related outcomes measures; VRS: verbal rating scale; CPIP: chronic postoperative inguinal pain.

At the second follow-up questionnaire (Table 7), pain levels were almost the same as those reported before. No significant differences were found between the three studied groups.

Overall the late results were similar across the three studied groups, specifically with regard to the rates of identified recurrence (approximately 0.6%) and severe chronic postoperative inguinal pain (CPIP < 1%).

## Discussion

In this large series of 3,043 “non-giant” scrotal hernia repairs, prospectively registered and followed up for 2 years, the studied techniques resulted in similar late results marked with a very low rate of both identified recurrences and chronic postoperative inguinal pain (CPIP). Each technique had its benefits and drawbacks. Of these, TIPP appeared to be a feasible, safe and effective method of repairing scrotal hernias. Having a choice of effective techniques may help to tailor the treatment of these particular types of groin hernia.

The global results of the entire series included a very low (less than 2%) rate of serious (Clavien-Dindo >/= III) postoperative complications (Table 6), a low rate of identified recurrences, and a low rate of late complications (e.g., late infection/mesh removal), at a median follow-up period of 2 years or more (Table 7).

From the patients’ perspective the repair of scrotal hernias resulted in a considerable reduction in pain or discomfort and a clear improvement in their quality of life (QoL), in both the 30-day postoperative outcomes (Table 5) and in the late follow-up. When comparing the preoperative PROMs (Table 3) with the late postoperative PROMs (Table 7), the relevant (moderate + severe) VRS pain considerably decreased from 43.8%, 36.1%, 24.1% at baseline to 10.3%,6.6%, 3.0%, and 6.1% at the 2-Year follow-up, respectively for Lichtenstein, TIPP and laparoscopic repairs. Which is four to six times less. While the impact of preoperative pain forced patients to interrupt or give up some activities in approximately 20%–30% of cases, fewer than 1% assessed that their postoperative discomfort was more troublesome than their preoperative condition. The fact that patients with scrotal hernias may benefit the most from their surgery has already been underlined in the literature [1, 3].

Of course, the scrotal hernias included in this study are completely different from those encountered in low-resource countries, which are often of the S2 or S3 type according to the recently proposed classification [1]. Such giant hernias are at risk of many technical difficulties [19–21], including loss-of-domain issues [22]. Moreover, our exclusion criteria, (emergency surgery, recurrent hernias, suture repairs, Rives-Stoppa repairs and miscellaneous repairs), probably resulted in the exclusion of some of the most complex cases. On the other hand (Table 3), the patients included in the present study were elderly, with a median age ranging from 71 years (TEP/TAPP) to 75 years (Lichtenstein or TIPP), they had comorbidities with an ASA class of 3-4, ranging from 16.1% (TEP/TAPP) to 31.8% (Lichtenstein), and many were on anticoagulant therapies, especially in the Lichtenstein group (23.4%).

Similar patient characteristics were found in 2,710 scrotal hernias registered in the German Herniamed registry, published in 2021 [3]. This major study in the field of scrotal hernias, mainly focused on the pre-, intra- and postoperative results of scrotal hernias, compared to those of lateral and medial hernias. Additionally, the study compared the results of laparo-endoscopic versus open procedures. Unfortunately, the minimally invasive open techniques were not individualised, and the distribution of the techniques used in the scrotal group (TEP: 8.2%; TAPP: 20.5%; Lichtenstein: 64.3%) was far from the distribution observed in the present series (Table 1), which limits comparisons.

In the present study, the late results of TIPP/MOPP, TEP/TAPP and Lichtenstein were almost the same, with regard to identified recurrences (approximately 0.6%) and severe chronic postoperative inguinal pain (CPIP < 1%). Differences between groups were found in terms of demographics, intraoperative events and early postoperative outcomes. Each of the studied techniques showed some benefits and drawbacks, which will be discussed in the context of the literature albeit scarce in the case of scrotal hernias.

Laparoscopic repairs, used in this series both for less complex patients (lower number of ASA 3-4 patients and patients on anticoagulants) and hernias (lower rate of L3/M3 defects), provided better nerve preservation, shorter operating times, higher outpatient rates and lower early postoperative pain. Conversely laparoscopic repairs had a few more conversions and postoperative organ space complications than the open procedures.

Although very rare, and not statistically significant in this large series, these findings have also been reported in both common [23, 24] and scrotal hernia repairs: Bansal et al. [25], in a series of 144 large hernias (including 10 “massive”) in Indian patients, reported a conversion rate of 25% in TEP (17.6% converted to TAPP and 7.1% converted to open) and a conversion rate of 10.2% in TAPP (converted to open). These rates may appear high, but this is quite an old series as the first cases date back to 2004. In 2020, Morrell described the Primary Abandon-of-the-Sac (PAS) technique during TAPP [26]. Using this, he operated on 26 scrotal hernias without conversion. In our laparoscopic group, the hernia sac was incompletely resected in 84.4% of the cases. The rate of seromas and hydroceles was not higher in the laparoscopic group than in the open groups in which the hernia sac was completely resected in more than two-thirds of cases. Similarly, Nikolian et al [27] showed in their series, that the primary abandonment of the sac in the management of scrotal hernias did not result in seromas or haematomas requiring procedural intervention.

The recently published guidelines on scrotal hernia repair [1] state: *“TEP may be employed safely with expertise, but one should have low threshold to convert to TAPP or open if technically not feasible. TAPP is the safest MIS approach for irreducible scrotal*”. [SIC].

Regarding the TEP technique, Ferzli et al. suggested the systematic division of the epigastric vessels to facilitate the hernia sac reduction [28]. Six years later, they reported on 94 cases of TEP for large scrotal hernias with nine cases (9.5%) requiring conversion to an open procedure, three cases (3.2%) completed with a conventional open preperitoneal approach, whereas six patients (6.4%) underwent repair with a combined approach [29].

In the present series (Table 2), 19 (4.6%) TEPs were converted: 10 to TAPPs, 7 to Lichtensteins, 1 to TIPP and 1 to another mesh repair. Fifteen TAPP (1.2%) were converted to 5 Lichtenstein procedures, 7 to other mesh repairs and 3 to suture repairs. As stated in the International Endohernia Society’s update of TAPP and TEP guidelines [30], “*TEP inguinal-scrotal hernia repair remains an advantageous approach during the difficult scrotal hernia that requires ‘‘conversion’’ to an open repair, because the pre-peritoneal dissection performed laparoscopically allows for reduction of the hernia and optimal mesh placement once the hernia repair has been converted and is performed from the anterior approach*”. [SIC] Similar findings were found in the TEP conversions to TIPP registered in the present series.

Malazgirt et al. [31] suggested using the open posterior approach for large or complex inguinal hernias, as this facilitated the handling and repair of difficult hernias in their experience.

In our series, TIPP repairs had a low conversion rate, better nerve preservation than that of Lichtenstein repairs, the lowest rate of serious postoperative complications and the highest rate of day surgery. The hernia sac was completely resected in 64% of cases without injury to the spermatic cord or need for a unilateral orchidectomy.

As with laparoscopic repairs, the preperitoneal mesh did not require any fixation in almost all the cases (96.5%).

The feasibility, safety, and effectiveness of TIPP are well known for common groin hernia repairs [4, 32, 33], but needed to be demonstrated for scrotal hernias. This was the focus of this study.

The technical feasibility of TIPP in scrotal hernias, suggested (Table 1) by a similar frequency of use in the scrotal series (11.8%) and the entire series (11.9%), was confirmed by a very low rate (0.8%) of conversions (Table 2). Its safety in scrotal hernia repairs was shown by a very low rate of serious (Clavien-Dindo >/= III) postoperative complications. The effectiveness of TIPP in scrotal repair was demonstrated by a low prevalence of identified recurrences (0.6%) at a median follow-up duration of 59 months.

A common criticism of the TIPP approach is the need for dissection going on in both planes, thus virtually hampering a possible approach on a “virgin” plane [34]. This is true, but not as significant as it seems. As shown in this series, the recurrences after TIPP are rare. They can be fixed either with the TAPP technique or with an open approach [35], because in TIPP the pre-fascial inguinal dissection is not as extensive as what is required for the Lichtenstein technique.

The Lichtenstein technique was mainly used for the most comorbid and the most symptomatic patients (Table 3). It was also the most commonly used technique for large M3 or L3 defects and for “pantaloon” hernias (combining lateral and medial defects) (Table 4). It was associated with a high number of resections of the inguinal nerves and had the highest rate of mesh fixation. On the other hand, this technique was achieved with fewer intraoperative technical difficulties than in the preperitoneal techniques (Table 4). The present study showed that, probably due to preoperative tailoring, the Lichtenstein group significantly collected many of the most complex patients and hernias. The Lichtenstein technique can be used to treat giant inguinoscrotal hernias [21], while in these challenging cases other teams opt for a preperitoneal mesh, inserted via a para-rectal incision [20] or using a modified Rives technique, often combined with visceral or omentum resections and/or completed with component separation techniques [22]. According to our exclusion criteria, such hernias were not included in the present study (Figure 1). In our series, the Lichtenstein technique was not only a default technique but also a fallback procedure: 15 (40.5%) of the 37 conversions occurring in laparoscopic or TIPP techniques resulted in a Lichtenstein procedure (Table 2). On the other hand, 18 (1.7%) intended Lichtenstein repairs required a conversion, mainly to a suture repair. This confirms that scrotal hernia repair can be challenging, even in “expert” hands.

The guidelines on scrotal hernia repair [1] also state: “*Depending on expertise, minimally invasive techniques can safely be employed. Although laparoscopic options are feasible, open repair remains the default operation for irreducible scrotal hernias. It is suggested that surgeons treating scrotal hernias are proficient in both anterior and posterior approaches.* As seen in the Results section, the participating surgeons did not always use the same technique for all their patients, but rather tailored the technique according to the characteristics of their patients and the hernia and they were able to easily convert one technique into another.

In the present series no significant difference was found between groups regarding the recurrence rate (0.6%) or the prevalence of severe CPIP (less than 1%).

These results are in line with the recently published late evaluation of recurrences and groin pain 8 years after the TEPLICH RCT [36], which compared TEP and Lichtenstein techniques in common groin hernia repairs.

In TAPP repairs for scrotal hernia repairs, Leibl et al. [37] reported a recurrence rate of 1% at a 30-month follow-up in 191 TAPP. Some years later, the same team [38] performed an analysis of 440 scrotal hernias in a large series of 8,050 TAPP repairs. The overall recurrence rate was 0.7%, compared to 2.7% for scrotal hernias.

### Limitations

This study is not without limitations. This is an observational study of a registry and therefore selection bias could not be avoided. Furthermore, it was neither randomised nor propensity-score matched.

Matching on age, BMI, diabetes, and tobacco use was not mandatory because for these parameters, there were no statistical differences among the three groups. Conversely, matching on anticoagulant therapies would have concealed one main asset of open versus laparoscopic techniques and matching on ASA class or preoperative symptoms would have concealed the specific benefits of Lichtenstein as the default technique. Moreover, our aim was not to determine the superiority of one technique over another but rather to assess the feasibility, safety, and effectiveness of TIPP in scrotal repair.

Not all patients underwent clinical examination. Telephone follow-up is not the optimal way to monitor patients after inguinal hernia surgery, and therefore some subclinical hernia recurrences may have been missed. However, the methodology was the same for the three groups studied. Regular follow-up was performed using a formatted phone questionnaire, which is i) more convenient for a large number of patients (currently 65,000 in our registry), and much more efficient than postal/mail questionnaires/reminders [39, 40] ii) reliable to assess chronic pain and Q.O.L. in addition to detecting late events such as rehospitalisations, reoperations, bowel obstructions, and mesh infections.

The S1, S2, and S3 classifications of scrotal hernias [1] were published too recently to be implemented in the dataset of our registry, which was launched in 2011. Scrotal hernia was defined in the present study as an inguinal hernia that had descended into and caused any distortion of the scrotum. Giant hernias were not included in the present study (Figure 1). Thus, the external value of this study is limited to ‘non-giant’ scrotal hernias and to surgeons specialising in hernia surgery.

### Strengths

The strength of this study lies in the analysis of a large case series providing real-world data from a registry of high-volume surgeons evenly distributed over the studied techniques.

## Conclusion

In this study, the TIPP/MOPP, TEP/TAPP, and Lichtenstein techniques resulted in similar late results.

Their respective benefits are useful for tailoring the technique to the patient and their scrotal hernia.

This observational study assessed the feasibility and safety of the TIPP technique for scrotal hernia repair compared with other types of repairs (laparoscopic and Lichtenstein). The study shows that TIPP/MOPP is a feasible, safe and effective option for ‘non-giant’ scrotal hernia repair, yielding similar late results to those of the TEP/TAPP and Lichtenstein techniques. Thus, TIPP appears to be a valid alternative when the combined aims are to opt for a preperitoneal repair and a minimally invasive open route.

## Data Availability

The data analyzed in this study is subject to the following licenses/restrictions: Data from the Club Hernie registry available on demand. Requests to access these datasets should be directed to JG, jfgillion@wanadoo.fr.

## Footnotes

1
https://biostatgv.sentiweb.fr/
